# Microvesicle-Mediated Tissue Regeneration Mitigates the Effects of Cellular Ageing

**DOI:** 10.3390/cells12131707

**Published:** 2023-06-23

**Authors:** Nikolaos Panagiotou, Dagmara McGuinness, Armand M. G. Jaminon, Barend Mees, Colin Selman, Leon Schurgers, Paul G. Shiels

**Affiliations:** 1 Davidson Building, School of Molecular Biosciences, University of Glasgow, Glasgow G12 8QQ, UK;nikos.panagiotou@theracell.eu (N.P.); 2 School of Infection & Immunity, University of Glasgow, Glasgow G12 8QQ, UK; dagmara.mcguinness@glasgow.ac.uk (D.M.); 3 Department of Biochemistry, Cardiovascular Research Institute Maastricht, Maastricht University, Maastricht, 6229 ER Maastricht, NetherlandsThe Netherlands; 4 Department of Vascular Surgery, Maastricht University Medical Centre (MUMC), Maastricht, The Netherlands; barend.mees@mumc.nl; 5 Graham Kerr Building, College of Medical, Veterinary & Life Sciences, Institute of Biodiversity, Animal Health and Comparative Medicine, University of Glasgow, Glasgow G12 8QQ, UK; colin.selman@glasgow.ac.uk; 6 Institute of Experimental Medicine and Systems Biology, RWTH University Hospital, Aachen, Germany

**Keywords:** ageing, regenerative medicine, stem cells, extracellular vesicles, microvesicles, exosomes, wound healing

## Abstract

Extracellular vesicles (EVs), comprising microvesicles (MVs) and exosomes (Exos), are membranous vesicles secreted by cells which mediate the repair of cellular and tissue damage via paracrine mechanisms. The action of EVs under normative and morbid conditions in the context of ageing remains largely unexplored. We demonstrate that MVs, but not Exos, from Pathfinder cells (PCs), a putative stem cell regulatory cell type, enhance the repair of human dermal fibroblast (HDF) and mesenchymal stem cell (MSC) co-cultures, following both mechanical and genotoxic stress. Critically, this effect was found to be both cellular age and stress specific. Notably, MV treatment was unable to repair mechanical injury in older co-cultures but remained therapeutic following genotoxic stress. These observations were further confirmed in human dermal fibroblast (HDF) and vascular smooth muscle cell (VSMC) co-cultures of increasing cellular age. In a model of comorbidity comprising co-cultures of HDFs and highly senescent abdominal aortic aneurysm (AAA) VSMCs, MV administration appeared to be senotherapeutic, following both mechanical and genotoxic stress. Our data provide insights into EVs and the specific roles they play during tissue repair and ageing. These data will potentiate the development of novel cell-free therapeutic interventions capable of attenuating age-associated morbidities and avoiding undesired effects.

## 1. Introduction

The burden of age-related morbidities is expected to increase as a direct consequence of an expanding aged global population [1,2]. One’s capacity for tissue regeneration and wound healing diminishes with age [3]. Indeed, these processes are affected by the diseasome of ageing and are thus consequently impaired [4,5]. Mitigating the effects of the diseasome of ageing [6,7] is challenging, and a range of therapeutic interventions have been implemented to address this problem, including senolytic drugs designed to remove senescent cells from ageing or damaged tissue to restore normative physiological function [8,9,10]. The elimination of senescent cells by a range of senolytic agents has already been demonstrated to increase the health span and lifespan of mice [10,11,12,13]. However, it is unknown whether senolytic agents will function equivalently across one’s life course or how they will function in a multi-morbid milieu [14]. Alternative senotherapies are also in development. These include cellular therapeutics to restore or replace damaged tissue [15,16,17,18,19,20,21,22]. However, many of these cell-based therapies have technical and logistical hurdles associated with them, including an increased cancer risk, appropriate cell selection and differentiation, targeted delivery, costs, and scalability [23,24]. Such therapies have had varied success in practice, and their exact mechanism of action is not yet completely understood [25].

Notably, the recovery of tissue function following the administration of cell-based therapies has not typically been the result of the incorporation of cellular therapeutics directly into regenerated tissue [26], consistent with a model of paracrine-mediated tissue regeneration. Recently, this paracrine effect has been identified and characterised for Pathfinder cells (PC), a putative stem cell regulatory cell type that displays overlapping features with mesenchymal stem cells [27,28]. Rat PCs can stimulate the recovery of tissue structure and function across a species barrier in both concordant and discordant xenotransplant models of acute tissue damage, including streptozotocin (SZT)-induced damage to the pancreas and ischaemia/reperfusion damage to the kidney [27,28], with further studies consistently demonstrating a paracrine mode of action in mice [29].

Extracellular vesicles (EVs), including microvesicles (MVs) and exosomes (Exos), have been proposed experimentally as plausible candidates for facilitating paracrine-mediated tissue regeneration through the transfer of bioactive cargo, including proteins, mRNA, and miRNA [30,31], for both cellular and rodent models of organ damage [32,33,34]. Interestingly, several studies have provided evidence that links EVs with cell proliferation [35,36,37], migration [35,38,39,40], and angiogenesis [41,42] during wound healing. Nevertheless, an age-associated deterioration in one’s wound healing capacity has been well documented [43], and therefore cellular ageing might impair the efficacy of EV-mediated wound healing. However, the capacity of EVs to mediate wound repair has yet to be investigated in the context of cellular ageing. Additionally, the identity and nature of any EVs involved in tissue regeneration in vivo remain to be fully defined. Significantly, in an in vivo streptozotocin (STZ)-induced mouse model of diabetes, only MVs and not Exos were demonstrated to facilitate improved glycaemia [29]. The mechanistic basis of this remains unresolved. Consequently, understanding EV mechanisms of action during the maintenance of organismal homeostasis and tissue regeneration throughout one’s lifespan could prove beneficial for the development of novel senotherapies for the extension of human health span.

We have therefore investigated the therapeutic capacity of PC-derived EVs to facilitate tissue regeneration in the context of cellular ageing and disease. MVs and Exos were investigated and cross-compared for their capacity to facilitate repair in human dermal fibroblast (HDF) and mesenchymal stem cell (MSC) co-cultures of increasing cellular age, following mechanical stress induced by wounding and genotoxic stress induced by uraemic serum derived from patients with chronic kidney disease (CKD). In these tissue regeneration models, the effect of cellular ageing and underlying pathology were further investigated in HDF and human vascular smooth muscle cell (VSMC) co-cultures of both healthy and diseased abdominal aortic aneurysm (AAA) origins, with the VMSC and AAA cells derived from the same patient.

## 2. Materials and Methods

### 2.1. Extracellular Vesicle (EV) Isolation

Rat PCs were cultured for the purpose of isolating MVs and Exos. The PCs were initially plated on Corning^®^ T75 flasks (Merck, UK). Once the cells reached 70% confluency, they were transferred to bigger Corning^®^ T150 flasks (Merck, UK). When the PCs reached 70% confluency once again, they were used to prepare a 50 mL cell suspension in MV-free CMRL 1066 complete media. For this type of cell media, we used 10% heat-inactivated FBS that was centrifuged at 16,000× *g* for 3 h to remove MVs potentially present within it. The cell suspension was then transferred to a Corning^®^ HYPERFlask^®^ cell culture vessel (Merck, UK), which was then completely filled with MV-free CMRL 1066 complete media. When the PCs reached 70% confluency, the culture media was collected and immediately transferred to Corning^®^ 50 mL centrifuge tubes (Merck, UK) and centrifuged at 1000× *g* for 10 min to remove cell debris. The PC culture media supernatant was then transferred to a Corning^®^ Easy-Grip round plastic storage bottle (Merck, UK) and stored at 4 °C, before it was used for MV and Exo isolation.

The PC culture media were split into Corning^®^ 50 mL centrifuge tubes (Merck, UK) and centrifuged at 1000× *g* for 10 min to remove further cell debris. The supernatant was then carefully transferred to sterile Beckman Coulter^®^ tubes (Beckman Coulter Life Sciences, USA) and centrifuged at 16,000× *g* for 3 h at 4 °C in an Avanti J-25 centrifuge (Beckman Coulter Life Sciences, USA) with a JA-25.50 fixed-angle aluminium rotor (Beckman Coulter Life Sciences, USA). The supernatant was used for Exo isolation (see next paragraph), while the MV pellets were resuspended in 1 mL sterile PBS. All of the resuspended MVs were then transferred to a single Beckman Coulter^®^ tube, in order to concentrate the MV sample. The single tube was then filled completely with sterile PBS and was centrifuged again at 16,000× *g* for 3 h. The supernatant was discarded, and the MV pellet was resuspended in 1 mL sterile PBS. MV isolates were finally filtered twice with the use of MicroKros^®^ hollow fiber filters (Spectrum Laboratories, Inc., USA) 0.1 μm filters, to ensure removal of Exo contaminants. The MV isolates were finally stored at −20 °C.

The supernatant that contained the Exos was transferred to sterile Beckman Coulter^®^ tubes and centrifuged at 120,000× *g* for 3 h at 4 °C. The supernatant was removed, and the Exo pellets were resuspended in 1 mL sterile PBS. All the resuspended Exos were then transferred to a single Beckman Coulter^®^ tube, to concentrate the Exo sample. The single tube was then filled completely with sterile PBS and was centrifuged again at 120,000× *g* for 3 h. The supernatant was discarded, and the Exo pellet was resuspended in 1 mL sterile PBS. Exo isolates were then filtered twice through 0.1 μm Minisart^®^ syringe filters (Sartorius AG, Germany) to remove MV contaminants, which are larger than 0.1 μm in size. The Exo isolates were finally stored at −20 °C.

### 2.2. Cell Culture

The cells used in this study were adherent and cultured in Corning^®^ T75 flasks (Merck, UK) at 37 °C in a humidified incubator atmosphere maintained at 5% CO_2_. For passaging, the cells were washed twice with phosphate buffered saline (PBS) (Thermo Fisher Scientific, UK) and trypsinised at 37 °C for 5 min with Trypsin-EDTA (0.05%) (Thermo Fisher Scientific, UK). Complete cell media were used to deactivate the Trypsin, and the cells were then seeded with a ratio of 1:3 in new Corning^®^ T75 flasks.

### 2.3. Pathfinder Cells (PCs)

Rat PCs of pancreatic origin were cultured in CMRL 1066 medium with no glutamine (Thermo Fisher Scientific, UK) supplemented with 10% heat-inactivated fetal bovine serum (FBS) (Thermo Fisher Scientific, UK), 1% penicillin-streptomycin (Thermo Fisher Scientific, UK), 1% Amphotericin B (fungizone^®^) (Thermo Fisher Scientific, UK), and 1% GlutaMAX™ (Thermo Fisher Scientific, UK). The PCs originated from pancreatic tissue of 12-month-old Albino Swiss (Glasgow) rats. Their cytotype has been described extensively (28, 72). The pancreatic tissue was minced prior to culturing in serum-free medium, and the PCs emerged as a confluent monolayer after approximately 5 weeks in culture.

### 2.4. Human Dermal Fibroblasts (HDFs)

HDFs were cultured in Dulbecco’s modified Eagle’s medium (DMEM) with GlutaMAX™-I (Thermo Fisher Scientific, UK), supplemented with 10% heat-inactivated FBS (Thermo Fisher Scientific, UK), 1% penicillin-streptomycin (Thermo Fisher Scientific, UK), and 1% Amphotericin B (fungizone^®^) (Thermo Fisher Scientific, UK).

### 2.5. Mesenchymal Stem Cells (MSCs)

Human MSCs were cultured in MSCBM™ mesenchymal stem cell basal medium (MSCBM hMSC Basal Medium) (Lonza, UK) with necessary supplements (MSCGM hMSC SingleQuot Kit) (Lonza, UK). According to the manufacturer, the serum that was used neither promotes spontaneous differentiation of cells nor inhibits cell differentiation to osteogenic, chondrogenic, or adipogenic lineages when properly stimulated.

### 2.6. Vascular Smooth Muscle Cells (VSMCs)

Collection, storage, and use of tissue and human aortic samples were in agreement with the Dutch Code for Proper Secondary Use of Human Tissue. VSMCs were cultured in Medium 199 (Thermo Fisher Scientific, UK), supplemented with 10% heat-inactivated FBS (Thermo Fisher Scientific, UK), 1% penicillin-streptomycin (Thermo Fisher Scientific, UK), and 1% Amphotericin B (fungizone^®^) (Thermo Fisher Scientific, UK) and 1% GlutaMAX™ (Thermo Fisher Scientific, UK). The VSMCs originated from patients who had suffered a non-genetic abdominal aortic aneurysm. Specifically, the VSMCs were isolated by a surgeon from healthy and aneurysmal aorta of the same patient.

### 2.7. Co-Cultures

To prepare the co-cultures, cell suspensions in complete DMEM were prepared for HDFs and MSCs or VSMCs. HDFs and MSCs or VSMCs were counted, and then plated as a co-culture, at a ratio of 5:1 HDFs to MSCs or HDFs to VSMCs. Co-culturing, wounding, uraemic treatment protocol, and EV concentrations are described in the relevant method sections below or in the Supplementary Materials.

### 2.8. Effect of EVs Relative to Cell Age

EVs were derived from young PCs, passages 1–4 (with 1–12 cell duplications, respectively) and from old PCs, passage 17 (51 cell duplications) and tested on HDF–MSC wound healing assays (Figure S8).

### 2.9. Flow Cytometry

A series of drops using the Flow Cytometry Sub-Micron Size Reference Kit (Life Technologies, UK) with bead sizes of 0.2 µm, 0.5 µm, 1 µm, and 2 µm were added to 1 mL of PBS in 5 mL round-bottom polystyrene test tubes (BD Biosciences, UK) for flow cytometry and were run on a BD FACSVerse™ (BD Biosciences, UK) flow cytometer. A total of 200 μL of the EV isolates was added directly into flow cytometry tubes and run on the flow cytometer. The BD FACSuite™ (BD Biosciences, UK) software was used for the purposes of system setup, data acquisition, initial analysis, and shutdown. The data that were collected were analysed with the use of FlowJo^®^ Version 10.1 (FlowJo LCC, USA).

### 2.10. Senescence-Associated β-Galactosidase (SA β-gal) Assay

The cells were stained for SA β-gal 72 h after plating 50,000 cells per well, in 6-well plates. Initially, the Senescence Cells Histochemical Staining Kit (Merck, UK) was used according to the manufacturer’s guidelines. Briefly, the cell media were aspirated, and the cells were washed twice with PBS. The fixation buffer was then added, and the cells were incubated for 7 min at room temperature. The cells were washed 3 times with PBS, and 1.5 mL of the staining mixture (for 10 mL mixture: 8.5 mL milli-Q water, 1 mL staining solution, 125 μL Reagent B, 125 μL Reagent C, and 250 μL X-gal Solution, filtered through membrane with a pore size of 0.2 µm) was added. The cells were incubated in the staining mixture overnight at 37 °C without CO_2_ at a pH of 6 until they were stained blue. The cells were then washed three times with PBS and stained with 1.43 µM DAPI (Thermo Fisher Scientific, UK), diluted in PBS, at room temperature for 1 h on a moving platform and covered from light. The DAPI staining step was followed by three washes with PBS. The cells were finally covered with 2 mL of 70% glycerol solution diluted in milli-Q water. Visualisation was achieved with brightfield microscopy, and pictures were taken using a Zeiss Axio Observer microscope (Carl Zeiss Ltd., UK). Total cell number and cells positive for SA β-gal were scored. At least 1000 cells were counted per well.

### 2.11. γH2A.X Staining

The cells were stained for γH2A.X and DAPI 72 h after plating. The cell media were initially aspirated, and the cells were washed 2 times with PBS. The cells were then fixed with 100% methanol (Merck, UK) at room temperature for 5 min. The cells were washed another 2 times and permeabilised with 0.1% Triton™ X-100 (Merck, UK) diluted in PBS and incubated at room temperature for 5 min. The cells were washed another 2 times with PBS before addition of blocking solution. The blocking solution consisted of 1% bovine serum albumin, 10% goat serum, and 0.3 M glycine in 0.1% PBS-Tween^®^ 20 (all from Merck, UK). Incubation took place at room temperature for 5 min. The primary rabbit antibody anti-γH2A.X (phospho S139) (cat. No. ab2893, Abcam, UK) was added in a concentration of 1 µg mL^−1^, and the cells were incubated at 4 °C on a moving platform overnight. After removal of the primary antibody and 3 washes with PBS, the secondary goat anti-rabbit Alexa Fluor^®^ 488 antibody (1:1000; cat. no. ab150081, Abcam, UK) was added and allowed to bind at room temperature for 1 h on a moving platform. The secondary antibody was then removed, and the cells washed thrice with PBS. Finally, the cells were stained with 1.43 µM DAPI (Thermo Fisher Scientific, UK), diluted in PBS at room temperature for 1 h on a moving platform that was sheltered from light. The DAPI staining step was followed by three washes with PBS, and the cells were covered with 70% glycerol diluted in milli-Q water. Pictures were then taken with fluorescence microscopy using a Zeiss Axio Observer microscope (Carl Zeiss Ltd., UK). Total cell number and the number of cells expressing cytoplasmic chromatin fragment (CCF) events, i.e., γH2A.X positive in the cytoplasm, were recorded. At least 1000 cells were counted per well.

### 2.12. CDKN2A and CDKN1A Expression

RNA extraction: Cell samples (HDFs, MSCs, or VSMCs) were extracted before stress (wounding or uraemic serum addition) and 72 h after stress in 1 mL TRIzol™ reagent (Thermo Fisher Scientific, UK) with the use of a cell scraper. The samples were stored at -20 °C overnight. The samples were then centrifuged at 12,000× *g* for 10 min at 4 °C, and the supernatants were transferred to fresh tubes on ice. For every 1 mL of TRIzol™, 200 μL of chloroform (Thermo Fisher Scientific, UK) was added. Hence, 200 μL chloroform was used. The samples were mixed by hand for 15 s and incubated at room temperature for 3 min. They were then centrifuged at 12,500× *g* for 15 min at 4 °C. The aqueous phase was transferred to fresh tubes on ice. For one volume of aqueous phase, an equal volume of 95% ethanol (Merck, UK) diluted in milli-Q water was added.

The RNA Clean & Concentrator™-25 kit (Zymo Research, USA) was then used. Briefly, the samples were transferred to Zymo-Spin™ IIC columns in collection tubes and spun at 14,000× *g* for 30 s. After the flow-through was discarded, the column was pre-washed with 400 μL of RNA wash buffer and centrifuged at 14000× *g* for 30 s. The flow-through was then discarded. DNase I mix (75 μL DNA digestion buffer and 5 μL DNase I per sample) was added to each column, and the columns were then incubated at room temperature for 15 min. A total of 400 μL RNA Prep buffer was added, and the columns were centrifuged at 14,000× *g* for 30 s. The flow-through was discarded. Then, 700 μL of RNA wash buffer was added, and the columns were centrifuged at 14,000× *g* for 30 s. The flow-through was discarded. This step was repeated once more with 400 μL of RNA wash buffer which was centrifuged for 2 min this time. The flow-through was discarded, and each sample was transferred to a new Eppendorf tube. A total of 15 μL DNase/RNase-free water was added to each column and centrifuged at 14,000× *g* for 30 s. This step was repeated once more, and 30 μL of total flow-through was collected and placed on ice for 15 min.

Finally, 1.6 μL of the RNA extracts were used for RNA measurement with the use of the NanoDrop™ 2000 Spectrophotometer (Thermo Fisher Scientific, UK). The RNA samples were then stored at −80 °C.

RT-PCR: A total of 2.5 μL of random primers (3 µg μL^−1^) (Thermo Fisher Scientific, UK) and 2.5 μL of Deoxynucleotide mix (10 mM) (Thermo Fisher Scientific, UK) were added to 125 ng of RNA per sample. Nuclease-free water (Thermo Fisher Scientific, UK) was added to a final volume of 19 μL. The samples were mixed well by pipetting and incubated at 67 °C for 5 min. The tubes were then placed on ice before adding 16 μL of the prepared pre-mix, including 8 μL of 5× first-strand buffer, 2 μL of RNaseOUT™ recombinant ribonuclease inhibitor (40 U μL^−1^), 4 μL of 0.1M DTT, and 2 μL of SuperScript^®^ II Reverse Transcriptase (200 U μL^−1^) per well. The samples were then mixed well by pipetting.

qPCR: The qPCR mastermix was prepared following the manufacturer’s guidelines (Thermo Fisher Scientific, UK). Briefly, each reaction contained 5 μL of TaqMan™ Universal PCR mastermix II (no UNG), 3.5 μL of nuclease-free water, 0.5 μL of 20× TaqMan™ Assays qPCR primers (all from Thermo Fisher Scientific, UK) and 1 μL of product of RT-PCR reaction. Relative quantity of RNA was analysed using comparative threshold (Ct) method. Negative controls included a no-template control (no RNA at cDNA generation step) and an amplification control (no cDNA added).

### 2.13. Real-Time Cell Analysis (RTCA)

Wound healing and uraemic serum assays were performed in E-Plate VIEW 96 (ACEA Biosciences, Inc., USA), which is a specific type of 96-well plate, fused with gold microelectrodes that can detect the presence of adherent cells. Cell growth was recorded with the xCELLigence^®^ RTCA MP (ACEA Biosciences, Inc., USA). A total of 100 μL DMEM medium per well was added, and the plate was placed in the RTCA instrument in the incubator (37 °C, 5% CO_2_) to measure the background impedance. Additionally, a cell suspension in complete DMEM was prepared for HDFs and MSCs (or VSMCs). HDFs and MSCs (or VSMCs) were counted, and 5000 cells were plated as a co-culture, at a ratio of 5:1 HDFs to MSCs (or HDFs to VSMCs). The plate was filled to 200 μL per well with addition of the mixed cell suspension, and the cells were allowed to sediment for 30 min in the tissue culture hood. The plate was then placed in the RTCA instrument in the incubator for overnight incubation. Twenty-four hours later, after allowing for overnight attachment, a scratch wound was performed with a 20 μL sterile pipette tip for each well for the wound healing assays. For the uraemic serum assays, the media were gently removed and replaced with 150 µL fresh MV-free DMEM media, 20 µL of uraemic serum (10% final concentration), and 30 µL MVs (or Exos) (0.1 ng µL^−1^ of total RNA). For control, the wells were replaced with media containing equal volume of PBS instead of MVs. The plate was returned to the RTCA instrument in the incubator, and wound closure was recorded during a three-day period. Wells containing no cells and wells containing only media or media with MVs were also used as controls.

Cell proliferation assays in the absence of stress were also performed in E-Plate VIEW 96, and cell growth was recorded with the xCELLigence^®^ RTCA MP. A total of 100 μL of MV-free DMEM medium per well was added, and the plate was placed in the RTCA instrument in the incubator (37 °C, 5% CO_2_) to measure the background impedance. Additionally, a cell suspension in MV-free DMEM media was prepared for HDFs and MSCs. HDFs and MSCs were counted, and 5000 cells were plated as a co-culture, at a ratio of 5:1 HDFs to MSCs. The plate was filled to 200 μL per well with addition of the mixed cell suspension and MVs (0.1 ng μL^−1^) or a similar volume of PBS instead of MVs as a control. The cells were allowed to sediment for 30 min in the tissue culture hood. The plate was then placed in the RTCA instrument in the incubator, and cell proliferation was monitored for 72 h. Wells containing no cells and wells containing only media or media with MVs were also used as controls.

Cell migration assays in the absence of stress were performed in CIM-Plate 16 (ACEA Biosciences, Inc., USA), which is a specific type of 16-well plate comprising an upper chamber with a microporous membrane (8 μm pore diameter) and a bottom chamber. Gold electrodes on the underside of the upper membrane detect the presence of cells migrating through the pores. Cell migration was recorded with the xCELLigence^®^ RTCA DP (ACEA Biosciences, Inc., USA). The bottom chamber of the 16-well plate was completely filled with MV-free DMEM. The upper chamber was locked on top of the bottom chamber, and it was filled with 50 μL of MV-free DMEM medium. The fully assembled plate was placed in the RTCA instrument in the incubator (37 °C, 5% CO_2_) to measure the background impedance. A cell suspension in MV-free DMEM was prepared for HDFs and MSCs. Cells were then counted and the two cell types were mixed at a 5:1 ratio of HDFs to MSCs. A total of 5000 cells were then added to the upper chamber at a total volume of 170 μL per well, along with 30 μL of MVs (0.1 ng μL^−1^) or a similar volume of PBS instead of MVs as a control. Cells were allowed to sediment for 30 min in the incubator. The plate was then placed in the RTCA instrument in the incubator, and cell migration was monitored for 72 h. This experiment was also repeated with addition of a similar dose of MVs (or PBS as a control) at the bottom chamber. Wells containing no cells and wells containing only media or media with MVs were also used as controls.

Data of the RTCA normalized cell index over time were collected and analysed with the use of a slope analysis by RTCA software, version 1.2.1 (ACEA Biosciences, Inc., USA).

### 2.14. 5-Bromo-2′-Deoxyuridine (BrdU) Labelling

Stress assays (wounding or uraemic serum addition) were performed in Corning^®^ 6-well plates (Merck, UK). The HDFs, MSCs, and VSMCs (healthy and aneurysmal) were counted and used immediately for plating on 6-well plates as monocultures. After allowing for overnight attachment in the incubator at 37 °C and 5% CO_2_, a scratch wound was performed with a cell scraper for the wound healing assays. The media were then gently removed and replaced with fresh MV-free DMEM media. For the uraemic serum assays, the media were gently removed and replaced with fresh MV-free DMEM media, 10% uraemic serum, and MVs. MVs were administered at a dose of 0.1 ng μL^−1^ of RNA per well. For control, the wells were replaced with MV-free media containing equal volume of PBS, instead of MVs.

The cells were stained with BrdU before stress and 72 h after stress. Initially, the 5-Bromo-2′-deoxyuridine Labelling and Detection Kit II (Roche, UK) was used as per manufacturer’s protocol. The cell media were aspirated, and the cells were washed twice with PBS before the BrdU labelling medium (1:1000 dilution in MV-free complete media, filtered through a membrane with a pore size of 0.2) was added for the labelling of DNA. The cells were incubated with the BrdU labelling medium at 37 °C for 30 min. They were then washed 3 times with washing buffer (diluted 1:10 in milli-Q water). The cells were then fixed in ethanol fixative (30 mL of 50 mM glycin solution and 70 mL absolute ethanol, with a pH of 2.0) for 10 min at −20 °C. The cells were then washed 3 more times with washing buffer, and the Anti-BrdU solution was added and allowed to act at 37 °C for 30 min. The cells were washed 3 times again, and the Anti-mouse-Ig-AP solution was added to the cells for 30 min at 37 °C. Finally, the cells were washed 3 times with washing buffer, and freshly prepared colour substrate buffer solution (100 mM Tris HCl, 100 mM NaCl, 50 mM MgCl_2_, at a pH of 9.5) was added at room temperature for 15 min before it was washed with washing buffer 2 times as well. The cells were then covered with 70% glycerol diluted in milli-Q water.

The cells were visualised with brightfield microscopy, using a Zeiss Axio Observer microscope (Carl Zeiss Ltd., UK). Total cell number and cells positive for BrdU were counted. At least 1000 cells were counted per well.

### 2.15. Time Lapse Fluorescence Microscopy

Wound healing assays were performed in Corning^®^ 6-well plates (Merck, UK). HDFs and MSCs were individually labelled with cell tracking dyes eBioscience™ CFSE (Thermo Fisher Scientific, UK) and PKH26 (Merck, UK), respectively. Optimised protocol was used for both cell types. After preparing serum-free cell suspension, the fluorescent cell tracking dyes were added at a final concentration of 5 μM for both dyes, and the cells were incubated in the dark at 37 °C for 10 min. Labelling was stopped by addition of 5 volumes of cold complete media and incubation on ice for a further 5 min. The cells were then washed 3 times with complete media to remove excess of the cell tracking dyes.

The cells were counted and used immediately for plating on 6-well plates as a co-culture with a 5:1 ratio of HDFs to MSCs. After allowing for overnight attachment in the incubator at 37 °C and 5% CO_2_, a scratch wound was performed with a cell scraper. The media were then gently removed and replaced with fresh MV-free DMEM media. MVs were administered at a dose of 0.1 ng μL^−1^ RNA per well. For control, the wells were replaced with MV-free media containing equal volume of PBS, instead of MVs.

Pictures were taken every 12 h over a 3-day period with fluorescence microscopy, using a Zeiss Axio Observer microscope (Carl Zeiss Ltd., UK). The number of cells migrating into the wound site during repair were counted.

### 2.16. Statistical Analysis

Comparisons were performed using GraphPad Prism and the most suitable method, which was either *t*-test or one-way ANOVA with Tukey’s or Dunnett’s test. Data are presented as means ± SD. *p* ≤ 0.05 was considered statistically significant. In figure legends, N corresponds to independent biological replicates of an experiment, and n corresponds to technical replicates.

## 3. Results

### 3.1. Only PC-Derived MVs and Not Exos Are Able to Accelerate Wound Healing

We explored the regenerative properties of PC-derived EVs to mediate wound healing in an HDF–MSC co-culture model, incorporating a scratch assay *in vitro*. The ageing characteristics of all of the cells used in subsequent studies, including senescence-associated (SA) β-galactosidase, cytoplasmic chromatin fragments (CCF), and CDKN2A and CDKN1A expression, are shown in Figure S1. All the biomarkers of ageing demonstrated an increase in expression with increasing cellular age. For the purposes of this investigation, co-cultures comprising HDFs and MSCs were employed to provide a basic surrogate for the tissue microenvironment [44]. Specifically, we investigated and cross-compared MVs and Exos for their therapeutic efficacy to facilitate wound repair. MVs and Exos were isolated from cell culture media of rat PCs through a series of sequential centrifugation and ultrafiltration steps [29]. They were then characterized by their size and surface markers. The MV isolates ranged between 0.1 and 1 μm in size, as expected [45], while the Exos were undetected via flow cytometry, as a consequence of their significantly smaller size and the triggering threshold of the instrument (Figure S2). We have previously shown Integrin β1, CD40, Rab5b, and CD63 to be expressed on these MV isolates, while CD9 was found to be expressed only in Exo preparations [29], as others have also reported [46,47].

**Figure 1:**
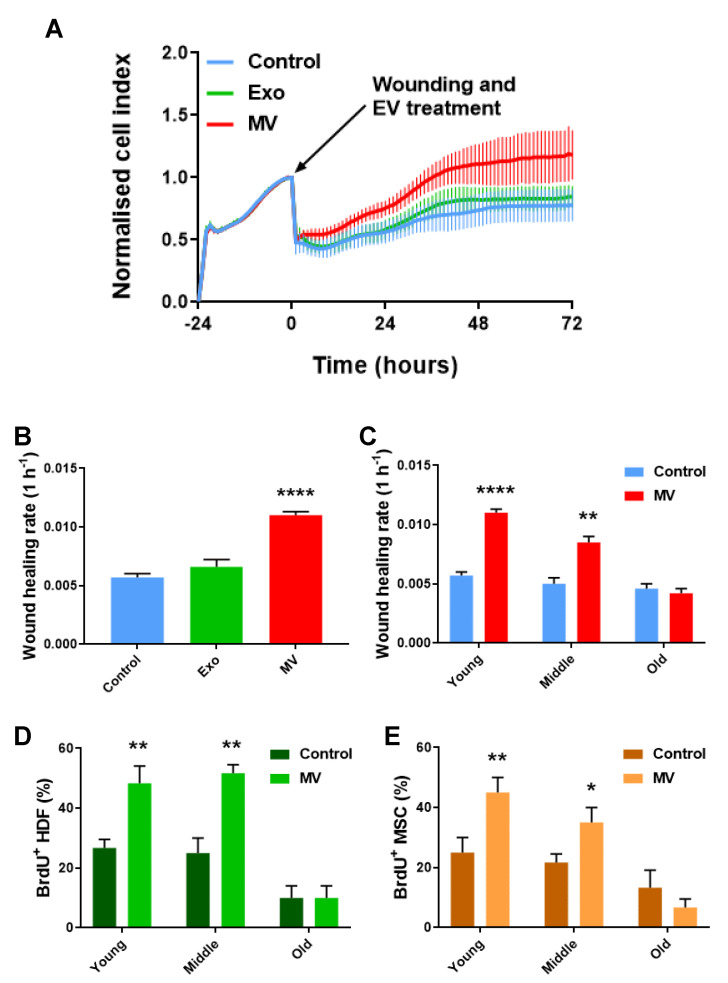
MV treatment improves cellular proliferative capacity following wounding, while old cellular age inhibits the MVs’ beneficial effect. (**A**) RTCA proliferation assay of a wound healing assay on a young HDF–MSC co-culture. Data are presented as mean ± SD (n = 6). (**B**) Wound healing rate of young HDF–MSC co-cultures treated with MVs and Exo, from 0 to 72 h. Data are presented as mean ± SD using one-way ANOVA with Tukey’s test (N = 3). (**C**) Wound healing rate of HDF–MSC co-cultures of increasing cellular age, after wounding and under MV treatment. Data are presented as mean ± SD; *t*-test (N = 3). (**D**) Percentage of BrdU^+^ HDFs of increasing cellular age, after wounding and under MV treatment. Data are presented as mean ± SD; *t*-test (N = 3). (**E**) Percentage of BrdU^+^ MSCs of increasing cellular age, after wounding and under MV treatment. Data are presented as mean ± SD; *t*-test (N = 3). * *p* < 0.05, ** *p* ≤ 0.01, **** *p* ≤ 0.0001.

We monitored wound repair with real-time cell analysis (RTCA) proliferation assays over a three-day period following wounding. The administration of Exos directly after wounding had no significant beneficial effect on wound healing, while the addition of MVs significantly enhanced the efficiency of cells in proliferating in response to wounding. Additionally, the MV-treated co-cultures were capable of complete wound closure, whereas the Exo treatment and non-treated controls failed to achieve this (Figure 1A,B).

### 3.2. MV-Mediated Wound Healing Capacity Declines in HDF–MSC Co-Cultures with Increased Cellular Age

The individual cell types within the co-cultures were characterized by age (passage number) as young (passages 1–4), middle-age (passages 8–12), and old (passages 16–20), respectively. Their proliferative capacity was then determined by an RTCA slope analysis in response to the mechanical stress (scratch wounds). Both the young and middle-age co-cultures displayed increased growth slopes following the addition of MVs and were able to completely close the wound over the course of the experiment. However, the older co-cultures were refractory to the MV treatment and were not able to fully close the wound over the 72-h time period of the experiment, equivalent to the untreated controls (Figure 1C). A further analysis indicated that the unassisted wound closure rate decreased with increased cellular age (Figure S3A), and this cellular age effect resulted in a significantly more rapid decrease in MV-mediated wound healing (Figure S3B,C). 5-bromo-2′-deoxyuridine (BrdU) staining was then employed to further confirm that any MV-associated increase in the wound closure rate was due to enhanced cell proliferation. The staining was performed individually for the HDF and MSC monocultures. The MV administration resulted in an increased number of actively proliferating HDFs and MSCs in the young and middle-age cultures. No difference was observed between the treated old HDFs, treated old MSCs, and controls (Figure 1D,E).

**Figure 2:**
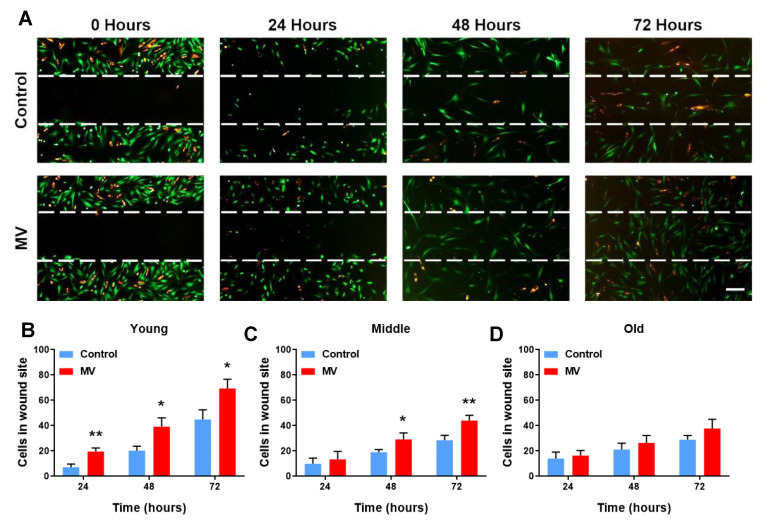
MVs improve the migration of HDFs and MSCs in the wound site but in an age-dependent manner. (**A**) Time-lapse fluorescence microscopy of a wound healing assay in a young HDF–MSC co-culture, treated with MVs. Wounding and MV administration took place at time point 0. HDFs labelled with CFSE (green) and MSCs with PKH26 (orange). Magnification 50×, scale bar 100 μm. Effect of MV administration on cell migration into the wound site; 24, 48, and 72 h after wounding in (**B**) young, (**C**) middle-age, and (**D**) old HDF–MSC co-cultures. Data are presented as mean ± SD; *t*-test (N = 3). * *p* < 0.05, ** *p* ≤ 0.01.

To further understand the potential processes through which MVs facilitate wound healing in HDF–MSC co-cultures, time-lapse fluorescence microscopy was utilized to observe the migration of these cells to the wound site, in response to the MV treatment during the wound’s closure. The HDFs and MSCs were differentially stained with CFSE and PKH26, respectively, and observed at 24-h intervals over a 3-day period. In the presence of the MVs, significantly more cells migrated to the site of mechanical damage, and this response was observed from as early as 24 h post wounding. The positive effect of the MV treatment also resulted in a higher number of cells after 48 and 72 h (Figure 2A,B). As the cellular age increased, the migratory efficacy of the cells diminished during MV-mediated wound healing. More precisely, in the middle-cellular-age co-cultures, a 24-h delay was observed in MV-mediated cell migration, comparing to that of the young co-cultures (Figure 2C). The old co-cultures were non-responsive to the treatment (Figure 2D).

The responses of each of the two co-cultured cell types to the MV treatment during the wound closure were then studied individually. The MV addition had a positive effect on young the HDFs, increasing cell migration into the wound site from 24 h onwards (Figure S4A). This response was delayed in middle-cellular-age HDFs and significantly more HDFs migrated to the wound site from 48 h onwards (Figure S4B). Finally, no differences were detected in the number of HDFs that migrated to the site of damage at an old cellular age (Figure S4C). Similarly, the young MSCs responded in a positive manner to MV administration and migrated to the wound site at 24, 48, and 72 h (Figure S4D). In the middle-cellular-aged MSCs, as in HDFs, the MVs’ positive effects, in terms of cell migration, were only apparent from 48 h onwards (Figure S4E). Finally, there were no observable differences in the number of cells that migrated to the site of damage, between the old MV-treated MSCs and the controls (Figure S4F).

Overall, these data suggest that MVs have the capacity to enhance the migration rate of both HDFs and MSCs in response to mechanical stress and repair wounds in vitro. This effect occurred in a time-dependent manner that was negatively affected by cellular ageing. Explicitly, the therapeutic effect was delayed in middle-age co-cultures and not observed in old co-cultures. Hence, these observations further validate that cellular ageing has a negative impact on the MV-mediated repair of mechanical stress. Interestingly, MVs did not increase the proliferative or migratory capacity in the control unwounded HDF–MSC co-cultures of increasing cellular age (Figure S5), indicating that they enhance cell proliferation and migration only in the presence of cellular damage.

### 3.3. Cellular Age Does Not Impact MV Efficacy under Morbid Conditions

**Figure 3:**
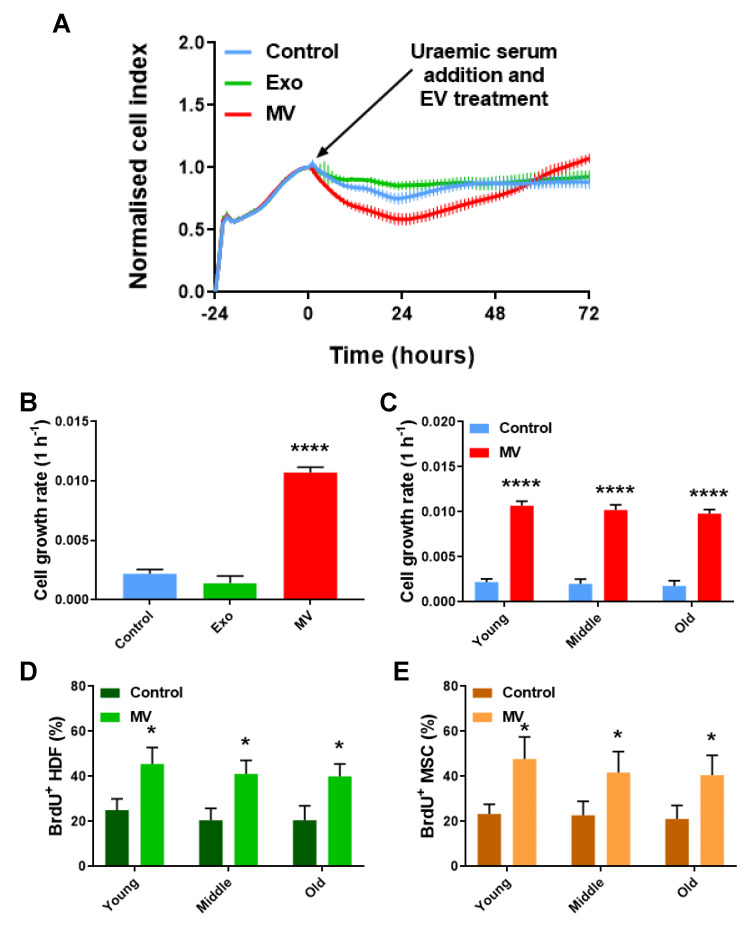
MV treatment improves cellular proliferation following uraemic stress, irrespective of cellular age. (**A**) RTCA proliferation assay of a genotoxicity assay on a young HDF–MSC co-culture. Data are presented as mean ± SD (n = 6). (**B**) Cell growth rate of young HDF–MSC co-cultures treated with MVs and Exos, from 0 to 72 h. Data are presented as mean ± SD; one-way ANOVA with Tukey’s test (N = 3). (**C**) Cell growth rate of HDF–MSC co-cultures of increasing cellular age, after genotoxic stress and under MV treatment. Data are presented as mean ± SD; *t*-test (N = 3). (**D**) Percentage of BrdU^+^ HDFs of increasing cellular age, after genotoxic stress and under MV treatment. Data are presented as mean ± SD; *t*-test (N = 3). (**E**) Percentage of BrdU^+^ MSCs of increasing cellular age, after genotoxic stress and under MV treatment. Data are presented as mean ± SD; *t*-test (N = 3). * *p* < 0.05, **** *p* ≤ 0.0001.

As premature ageing is a common feature of the diseasome of ageing, including CKD [48,49], we sought to determine if the features of morbidity (i.e., uraemia) affected MV efficacy in the context of cellular age. Co-cultures comprising HDFs and MSCs were employed to assess the regenerative properties of PC-derived MVs and Exos in facilitating the repair of genotoxic stress caused by uraemia derived from CKD stage-3 patients. During the first 24 h following the addition of uraemic serum, there was a distinct period of decreased cell growth. Interestingly, the RTCA data suggested that MV administration resulted in a significant decrease in cell growth, in comparison to both the control and Exo treatment, during this period of genotoxicity (Figure 3A and Figure S6A). Nevertheless, over the subsequent 48 h, an increase was observed in cell growth for the MV-treated samples. This time window was termed as a *healing period* because the cells were able to recover from the genotoxic stress and proliferate. During the healing period, the data indicated that MV addition had a positive effect on cell growth, while the control cell index did not significantly increase. In the control, this is indicative of a cytostatic effect in response to the presence of uraemic serum. Conversely, MVs were subsequently able to facilitate a positive response, and the cell growth significantly surpassed that of the control by the end of the experiment (Figure 3A). During the healing period, the slope analysis indicated that the administration of MVs significantly enhanced the rate of cell growth (Figure 3B). This was also true for the co-cultures of all the cellular ages that were investigated. Hence, the MV-associated therapeutic effect was not only recorded in young and middle-cellular-age mixed cultures, as was the case with the wound healing assays, but also in co-cultures of an old cellular age (Figure 3C). Furthermore, the increased MV-mediated cell loss during the genotoxicity period was also observed at all cellular ages (Figure S6B). The administration of Exos directly after the induction of genotoxicity had a protective effect during the initial 24-h genotoxicity period. The Exo-treated samples retained a higher cell index and significantly reduced cell loss compared to both the untreated controls and the MV-treated samples (Figure 3A and Figure S6A). Nevertheless, there was no significant Exo-associated effect during the healing period. The cell growth rate remained at similar levels to that of the untreated controls and at significantly lower levels to the MV-treated samples (Figure 3B).

These data are consistent with the MV treatment being a senotherapeutic. MVs may thus act as a putative senolytic agent in these assays, initially enabling the removal of senescent cells resulting from morbid conditions and subsequently promoting the repair of damaged tissue. This hypothesis builds upon previous studies that have demonstrated the ability of apoptotic cells to induce proliferation in neighbouring cells through compensatory proliferation signaling [50,51], mediated by the production and release of MVs that stimulate proliferation [52]. Other studies have also indicated that MVs are responsible for inducing apoptosis and cell proliferation following stress stimuli [53,54,55]. Thus, PC-derived MVs may play a key role in assisting and enhancing compensatory proliferation signaling following uraemic stress, initially inducing cell loss and subsequently increasing cell proliferation.

### 3.4. MV Treatment in the Context of Cellular Abnormality

Senotherapeutic strategies, such as those employing senolytic drugs, enable the removal of senescent cells for the purposes of improving age-related physiological functional capacity, as a means of extending one’s health span [9,11,56]. Senolytic activity, however, could have an adverse consequence, as a result of the removal of senescent cells whose presence is essential for maintaining tissue integrity. For instance, human vascular smooth muscle cells (VSMCs) in aneurysmal tissues have been identified to comprise populations of senescent cells [57,58,59,60,61,62], and their removal could hypothetically result in an undesirable aneurysmal rupture with dire physiological consequences. An abdominal aortic aneurysm (AAA) is an important health problem with a prevalence of up to 9% in adults over the age of 65 [63,64,65]; thus, the risk of AAAs in response to senotherapies merits further investigation. Here, we assessed the effect of MV administration to mediate repair in the presence of comorbidity, associated with increased levels of cellular senescence. For this reason, AAA VSMCs were compared with healthy VSMCs derived from the same patient and co-cultured with HDFs, due to their ability to repair mechanical and genotoxic stress in vitro, in response to MV treatment and with respect to cellular ageing.

**Figure 4:**
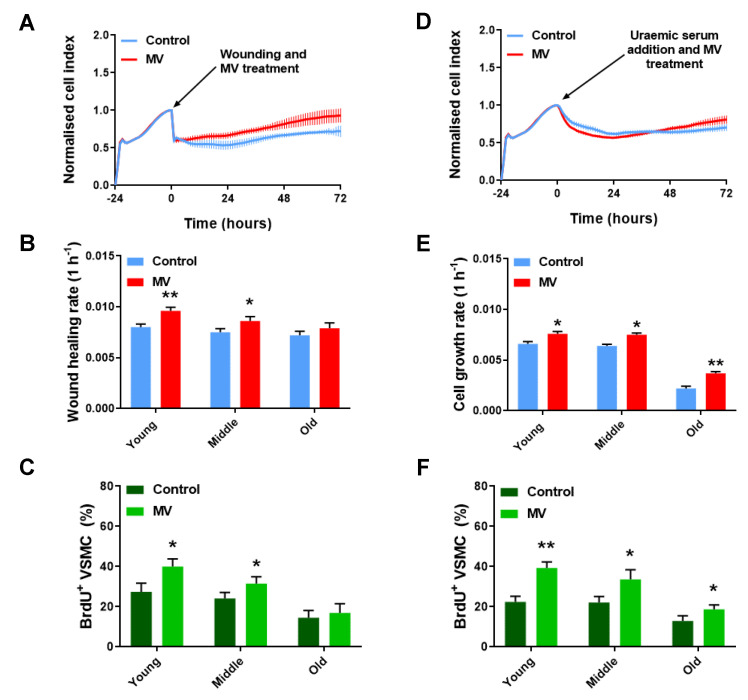
MVs improve the proliferation of HDF–VSMC co-cultures in response to wounding and uraemia in a stress- and age-dependent manner. (**A**) RTCA proliferation assay of a wound healing assay on a young HDF–VSMC co-culture. Data are presented as mean ± SD (n = 6). (**B**) Wound healing rate of HDF–VSMC co-cultures of increasing cellular age, after wounding and under MV treatment. Data are presented as mean ± SD; *t*-test (N = 3). (**C**) Percentage of BrdU^+^ VSMCs of increasing cellular age, after wounding and under MV treatment. Data are presented as mean ± SD; *t*-test (N = 3). (**D**) RTCA proliferation assay of a genotoxicity assay on a young HDF–VSMC co-culture. Data are presented as mean ± SD (n = 6). (**E**) Cell growth rate of HDF–VSMC co-cultures of increasing cellular age, after genotoxic stress and under MV treatment. Data are presented as mean ± SD; *t*-test (N = 3). (**F**) Percentage of BrdU^+^ VSMCs of increasing cellular age, after genotoxic stress and under MV treatment. Data are presented as mean ± SD; *t*-test (N = 3). * *p* < 0.05, ** *p* ≤ 0.01.

For normal (i.e., non-aneurysmal) VSMC co-cultures, the addition of MVs significantly enhanced the efficiency of cells to proliferate in response to wounding, in comparison to the non-treated controls in young and middle-aged co-cultures (Figure 4A,B). However, the addition of MVs did not have an observable effect on the wound closure rate in old mixed cell cultures. These co-cultures were refractory to the MV treatment during the 72 h of the experiment (Figure 4B). The actively proliferating healthy VSMCs (BrdU positive) were quantified, and it was observed that the MV administration resulted in complete wound closure and an increased number of actively proliferating cells, in comparison to that of the untreated controls. This was only true for young VSMCs, while middle-age and old-age VSMCs showed no difference from the controls (Figure 4C).

In uraemic genotoxicity assays, MV administration resulted in a significant decrease in the cell index (thus reduced cell growth), relative to that of the controls during the genotoxicity period from 0 to 24 h after the uraemic serum addition. Following the genotoxicity period, an increase was detected in the cell index in the MV-treated samples. Specifically, the cell index surpassed that of the untreated controls. Hence, the elevated cell growth during the healing period from 24 to 72 h of the experiment was attributed to MV action, with these cells able to recover from the genotoxic stress and proliferate. The untreated controls were unable to recover from the same genotoxic stress, and after an initial reduction in the cell index (0–24 h), the cell growth remained unchanged from 24 h until the end of the experiment (Figure 4D). During the initial 24-h genotoxicity period, the slope analysis indicated that the presence of MVs significantly reduced the cell index and cell growth relative to that of the controls. Consequently, MV administration following genotoxicity appeared to increase cell loss in comparison to that of the non-treated controls. Cell loss was elevated as the cellular age of the co-cultured cells increased (Figure S7A). This is consistent with senescent cell accumulation with cellular age and their subsequent removal following repair after genotoxic damage. During the healing period, from 24 to 72 h, the slope analysis indicated that the administration of MVs significantly enhanced the rate of cell growth in all age classes, in comparison to that of the controls (Figure 4E). However, the rate of cell growth associated with the old cellular age was lower than that of the young- and middle-cellular-age co-cultures. Additionally, 72 h after the addition of uraemic serum, it was observed that the MV administration enhanced the number of healthy, actively proliferating BrdU-positive VSMCs compared to that of the untreated controls. This observation remained unchanged, irrespective of the cellular age in the assay (Figure 4F).

**Figure 5:**
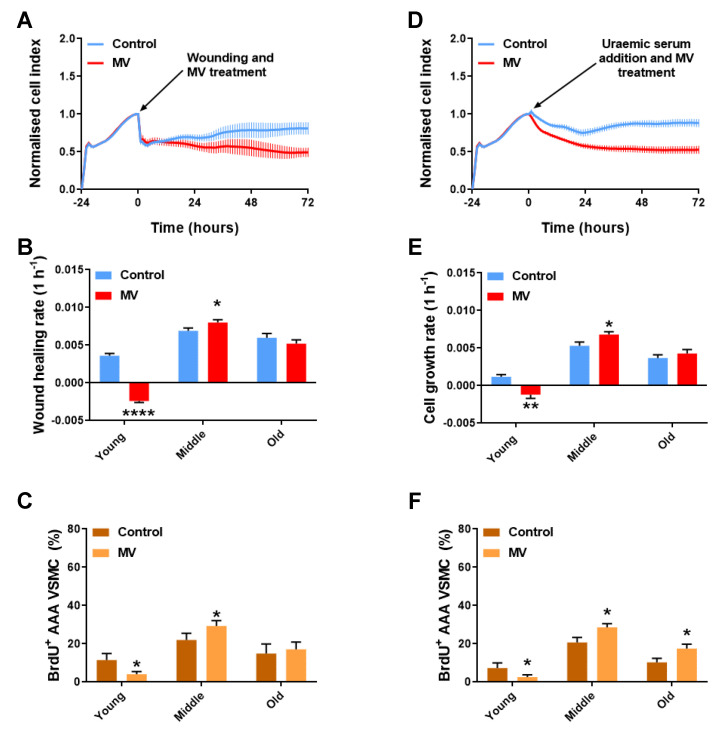
HDF-AAA VSMC co-cultures in response to MV treatment following wounding and uraemia. (**A**) RTCA proliferation assay of a wound healing assay on a young HDF-AAA VSMC co-culture. Data are presented as mean ± SD (n = 6). (**B**) Wound healing rate of HDF-AAA VSMC co-cultures of increasing cellular age, after wounding and under MV treatment. Data are presented as mean ± SD; *t*-test (N = 3). (**C**) Percentage of BrdU^+^ AAA VSMCs of increasing cellular age, after wounding and under MV treatment. Data are presented as mean ± SD; *t*-test (N = 3). (**D**) RTCA proliferation assay of a genotoxicity assay on a young HDF-AAA VSMC co-culture. Data are presented as mean ± SD (n = 6). (**E**) Cell growth rate of HDF-AAA VSMC co-cultures of increasing cellular age, after genotoxic stress and under MV treatment. Data are presented as mean ± SD; *t*-test (N = 3). (**F**) Percentage of BrdU^+^ AAA VSMCs of increasing cellular age, after genotoxic stress and under MV treatment. Data are presented as mean ± SD; *t*-test (N = 3). * *p* < 0.05, **** *p* ≤ 0.0001.

In the AAA VSMC co-cultures, the addition of MVs significantly reduced the efficiency of the cells to proliferate in response to wounding, in comparison to that of the controls (Figure 5A). The slope analysis from 0 to 72 h indicated that the proliferative capacity of young-cellular-aged co-cultures, in response to mechanical stress, was significantly decreased after the addition of MV. Additionally, these co-cultures retained a negative slope over the 72-h period and did not display signs of recovery. These data suggest that the addition of MVs resulted in cell loss. However, middle-cellular-age co-cultures were able to respond positively to MV administration and the rate of wound repair was significantly enhanced, in comparison to the controls, while the addition of MVs in old co-cultures did not have an observable effect on the rate of wound closure (Figure 5B). Three days post wounding, BrdU staining indicated that the MV administration resulted in reduced cell proliferation in the young AAA VSMC cultures. Nonetheless, complete wound closure and an increased number of actively proliferating cells were observed with middle-age AAA VSMC cultures, in comparison to the untreated controls. We hypothesised that through the cell culture and continuous passaging of cells, non-senescent cell selection happened naturally, and thus senescent cells numbers dropped, as confirmed by the significantly lower levels of SA β-gal and CCF that were observed in the middle-age AAA VSMCs, compared to the young, highly senescenct AAA VSMCs (Figure S1A,B). Finally, the MV-treated old-cellular-aged cultures from the AAA-derived cells were indistinguishable from the controls (Figure 5C), indicating that proliferative senescence occurred (Figure S1), and hence an increased cellular age was a factor in cells becoming refractory to the treatment.

In the genotoxicity assays, the addition of MVs had a significant negative impact on cell growth (Figure 5D). The slope analysis from 0 to 24 h indicated that the proliferative capacity of young co-cultures, in response to genotoxic stress, was significantly decreased after the addition of MVs (Figure S7B). Interestingly, the young co-cultures retained a negative slope over the 72-h period and did not display any signs of recovery, while the middle-aged co-cultures that were treated with MVs were able to respond positively to MV administration and recover by the end of the experiment, through a significant increase in cell growth compared to that of the untreated controls. No effect was observed in the old-cellular-age co-cultures (Figure 5E). In the AAA VSMC mono-cultures, a quantification of BrdU-positive cells revealed that the MVs decreased the ability of the cells to proliferate in the presence of uraemic serum at a young cellular age. Nevertheless, MV administration was associated with an increased percentage of actively proliferating cells in middle- and old-cellular-age AAA VSMCs (Figure 5F).

## 4. Discussion

Due to population ageing, there is an ongoing demographic shift which will lead to a significant increase in the number of elderly individuals around the world [66]. This represents a major societal and global health challenge with massive economic implications, due to the elevated burden of age-related morbidities that is estimated to increase accordingly [1,2,67]. Thus, measures are sought to improve one’s health span and increase the number of years of healthy old age [32]. The ageing trajectory can be altered by genetic, dietary, and pharmacological interventions, as demonstrated in model organisms [68,69,70,71]. However, the likelihood that these interventions can be directly translatable in humans is limited by unwanted side-effects or because they are impractical to undertake [68]. Consequently, there is a need to identify novel, safe, and practical interventions that ultimately improve late-life health in humans.

Therapeutic strategies that activate relevant repair processes in severely or terminally non-functional tissue are arguably the best approach for dealing with age-related pathophysiology [32]. Cell-based therapies, using a number of different cell types, such as induced pluripotent stem cells (iPSCs) [15,16], mesenchymal stem cells (MSCs) [17,18,19,20], very small embryonic-like stem cells [21,22], Pathfinder cells (PCs) [27,28,72], and others, are actively being developed [73,74]. Nevertheless, they are beset by logistical, technical, and ethical hurdles [23,24]. These therapies have had varied success and their exact mechanism of action remains poorly understood. Critically, the recovery of tissue function by particular cellular therapies has typically been suggested to occur without the incorporation of cellular therapeutics directly into the tissue being investigated [26]. These observations are consistent with a paracrine-mediated tissue regeneration model. Extracellular vesicles (EVs) have been experimentally proposed as strong candidates for mediating such a paracrine effect, as they are involved in cell-to-cell communication [30,31]. EVs have previously been explored for their capacity to facilitate paracrine-mediated tissue regeneration in cellular and rodent models of organ damage [32,33,45,75,76,77,78]. Such therapeutic strategies that enable severely or terminally non-functional tissue to activate relevant repair processes are arguably the best approach for dealing with the age-related loss of one’s physiological functional capability [32].

In this study, we have demonstrated that only the microvesicle (MV) component of EVs derived from PCs demonstrate a regenerative capability and therapeutic efficacy to initiate and enhance the repair process in vitro, following both wounding and uraemic serum-induced genotoxicity. This observation is consistent with a previous report linking PC-derived MVs with tissue repair in vivo, in a model of streptozotocin (STZ)-induced diabetes [29]. While MVs were shown to be therapeutically superior, exosome (Exo) treatment was associated here with an anti-apoptotic effect following the uraemic serum addition. Although Exo action alone was not sufficient to enhance repair and its effects were short-term, acting together with MVs might prove to be an effective therapeutic strategy worth exploring in the future.

Additionally, our in vitro data indicate that MVs significantly enhance wound repair, associated with increased proliferation and the cellular migration of both HDFs and MSCs into the wound site following mechanical injury, but this effect was negatively affected by cellular ageing. Thus, an interplay has been indicated to exist between MV-mediated regeneration and ageing and appears to be context dependent. Specifically, ageing appeared to interplay and negatively impact the MV-mediated repair of mechanical, but not genotoxic, stress. This suggests that MV-facilitated repair processes deteriorate with age at a different rate with respect to the type of stress that induces cellular repair. Notably, MVs did not increase the proliferative or migratory capacity in the unwounded controls, irrespective of cellular age. Hence this effect is only observed in the presence of cellular damage.

Our data are consistent with a scenario in which MVs can act as senotherapeutic and senolytic agents, promoting cell loss and enabling the restoration of physiological homeostasis in damaged tissue. This is in keeping with previous studies that demonstrated the ability of MVs derived from apoptotic cells to induce proliferation in neighbouring cells through compensatory proliferation signalling [52,53,54,55]. This is also consistent with PC-derived MVs enabling compensatory proliferation signalling in the presence of uraemic stress. In the genotoxicity assays, we observed that MVs improved cellular growth responses and enhanced the recovery of aged cells, unlike when grown under normative conditions, or when subject to mechanical stress. Notably, the addition of MVs initially resulted in increased cell loss before enhancing cell growth, consistent with the hypothesis of an MVS-induced senolytic effect in which senescent cell removal is enabled.

This was further validated in AAA VSMCs, which were highly senescent, as others have also demonstrated for both abdominal and thoracic aortic aneurysms [57,58,59,60,79]. The MV administration led to significantly increased cell loss in the presence of both wounding and uraemic serum at a young cellular age. In contrast, the MV administration was associated with an increased percentage of actively proliferating cells following stress in both middle-age and old AAA VSMCs, in which the senescent cell presence was lower in comparison to that of the young cellular age. These findings suggest that an MV-associated putative senolytic effect could have negative implications in the presence of an underlying pathology, such as an AAA, that is associated with elevated levels of cellular senescence and in the presence of a secondary stressor (i.e., wounding or uraemia). As others have discussed recently with regards to senotherapies, a cautionary approach should be adopted to avoid adverse events [80], such as the one reported in this study. Overall, highly senescent populations of cells which have resulted from comorbidity, such as AAA VSMCs, are less capable of responding to subsequent stress, while MV-mediated regeneration demonstrates a deleterious effect, causing cell removal and a reduced capacity for tissue repair. These observations need to be further investigated in animal models of aneurysms and comorbid conditions, for which senescent cells play an essential role in maintaining tissue homeostasis, and their removal could putatively lead to undesirable consequences for the organism, such as an aneurysm rupture. Additionally, our data indicate that the exploration of MV payloads is essential to understanding the mechanistic basis of our observations and thus merits investigation.

Overall, under normative growth conditions, MVs appear inert with respect to their effects on cell proliferation and migration. However, they are therapeutically efficacious and show an age-dependent effect in the presence of mechanical wounding, but not in the presence of genotoxicity. Explicitly, MV treatment repaired middle-aged co-cultures at a slower rate and was unable to repair mechanical injury in old co-cultures; nevertheless, it was therapeutically efficacious following genotoxic stress. Thus, MV-mediated repair interplays with ageing in a stress-specific manner. The reasons for this are unclear. There is a significant paucity of information on the role of EVs in ageing, and our data help address some aspects of this, specifically with respect to cell type (primary versus regenerative) and how they behave in the face of exposome stressors (physical versus physiological stress). One benefit of such a therapeutic approach is that it minimises the series of risks associated with cell-based interventions, including the risk of oncogenesis and small-vessel blockages; additionally, it has less variability in terms of therapeutic entities, easier quality assurance and quality control for clinical usage, and improved scalability, logistics, and cost.

## Data Availability

All the data that support the figures and the other findings are available upon request to the corresponding author.

## Supplementary Materials

The following supporting information can be downloaded at: https://www.mdpi.com/article/10.3390/cells12131707/s1, Figure S1: Ageing markers track cellular ageing in vitro. (A) Percentage of SA β-gal+ cells with increasing cellular age. Data are presented as mean ± SD; One-way ANOVA with Dunnett’s test (N = 3). (B) Percentage of CCF+ cells with increasing cellular age. Data are presented as mean ± SD; One-way ANOVA with Dunnett’s test (N = 3). (C) CDKN2A expression with increasing cellular age. Data are presented as mean ± SD; One-way ANOVA with Dunnett’s test (N = 3). (D) CDKN1A expression with increasing cellular age. Data are presented as mean ± SD; One-way ANOVA with Dunnett’s test (N = 3). * *p* ≤ 0.05, ** *p* ≤ 0.01, *** *p* ≤ 0.001, **** *p* ≤ 0.0001; Figure S2: Size characterisation of PC-derived MVs. Size profile of PC MVs characterised by forward-scatter (FSC-A) vs. side-scatter (SSC-A) flow cytometry in comparison to a series of beads of known sizes, including 0.2 µm, 0.5 µm, 1 µm, and 2 µm; Figure S3: Wound closure rate with increasing cellular age. (A) Physiological wound closure rate in HDF–MSC mixed cultures of increasing cellular age. Data are presented as mean ± SD; one-way ANOVA with Tukey’s HSD test (N = 3). (B) Effect of MV administration during wound repair in HDF–MSC co-cultures of increasing cellular age. Data are presented as mean ± SD; one-way ANOVA with Tukey’s HSD test (N = 3). (C) Combined. MV administration (red). PBS administration instead of MV (blue). Data are presented as mean ± SD; *t*-test (N = 3). * *p* < 0.05, ** *p* < 0.01; Figure S4: Quantification of HDF and MSC migration into the wound site. Effect of MV administration on HDF migration into the wound site; 24, 48, and 72 h after wounding in (A) young, (B) middle, and (C) old HDF–MSC co-cultures. Data are presented as mean ± SD; *t* test (N = 3). Effect of MV administration on MSC migration into the wound site; 24, 48, and 72 h after wounding in (D) young, (E) middle, and (F) old HDF–MSC co-cultures. Data are presented as mean ± SD; *t*-test (N = 3). * *p* ≤ 0.05, ** *p* ≤ 0.01; Figure S5: The proliferative and migratory capacity of HDF–MSC co-cultures declines with cellular age, while MVs do not affect these processes in the absence of cellular damage. (A) RTCA proliferation assay on a young HDF–MSC co-culture. Data are presented as mean ± SD (*n* = 6). (B) Cell growth rate of HDF–MSC co-cultures with cellular age. Data are presented as mean ± SD; *t*-test (N = 3). (C) RTCA migration assay on a young HDF–MSC co-culture. Data are presented as mean ± SD (*n* = 6). (D) Cell migration rate of HDF–MSC co-cultures with cellular age. Data are presented as mean ± SD; *t*-test (N = 3); Figure S6: Rate of cell growth in HDF–MSC genotoxicity assays from 0 to 24 h. (A) Cell growth rate of young HDF–MSC co-cultures treated with MVs and Exos, 24 h after uraemic serum addition. Data are presented as mean ± SD; One-way ANOVA with Tukey’s test (N = 3). (B) Effect of MV administration during the first 24 h of genotoxic stress in HDF–MSC co-cultures of increasing cellular age. Data are presented as mean ± SD; *t*-test (N = 3). *** *p* ≤ 0.001, **** *p* ≤ 0.0001; Figure S7: Rate of cell growth in HDF–VSMC and HDF-AAA VSMC genotoxicity assays from 0 to 24 h. (A) Effect of MV administration during the first 24 h of genotoxic stress in HDF–VSMC co-cultures of increasing cellular age. Data are presented as mean ± SD; *t*-test (N = 3). (B) Effect of MV administration during the first 24 h of genotoxic stress in HDF-AAA VSMC co-cultures of increasing cellular age. Data are presented as mean ± SD; *t*-test (N = 3). ** *p* ≤ 0.01, *** *p* ≤ 0.001; Figure S8: Effect of young- versus old-cellular-age Pathfinder cell MV administration during wound repair in HDF–MSC co-cultures of increasing cellular age. MVs derived from young Pathfinder cells (passage 1–4 (cell duplications 1–12)) or from old Pathfinder cells (passage 17 (cell duplications 51)) were tested for their efficacy on HDF–MSC wound healing assays. No differential effects on efficacy were observed relative to donor cell replication age. PBS was administered instead of MVs for controls. Data are presented as mean ± SD; One-way ANOVA with Tukey’s test (N = 3). ** corresponds to *p* < 0.01.
