# Supplementary material for: Microvesicle-Mediated Tissue Regeneration Mitigates the Effects of Cellular Ageing
Source: Cells. 2023 Jun 23;12(13):1707. doi: 10.3390/cells12131707 (PMC10340655; doi:10.3390/cells12131707)
Supplement: Supplementary file 1 [file cells-12-01707-s001.zip › cells-2456770-supplementary.pdf]

Supplementary Materials:

Supplementary Figures

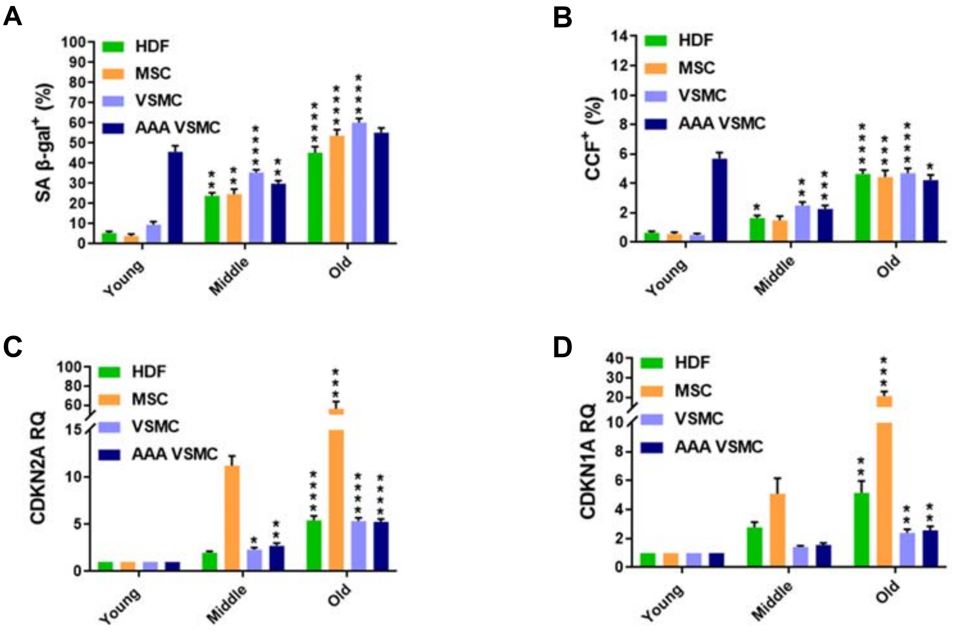

Figure S1. Ageing markers track cellular ageing *in vitro*. (**A**) Percentage of SA β-gal+ cells with increasing cellular age. Data are presented as mean ± SD; One-way ANOVA with Dunnett's test (N=3). (**B**) Percentage of CCF+ cells with increasing cellular age. Data are presented as mean ± SD; One-way ANOVA with Dunnett's test (N=3). (**C**) CDKN2A expression with increasing cellular age. Data are presented as mean ± SD; One-way ANOVA with Dunnett's test (N=3). (**D**) CDKN1A expression with increasing cellular age. Data are presented as mean ± SD; One-way ANOVA with Dunnett's test (N=3). *$p \leq 0.05$, **$p \leq 0.01$, ***$p \leq 0.001$, ****$p \leq 0.0001$.

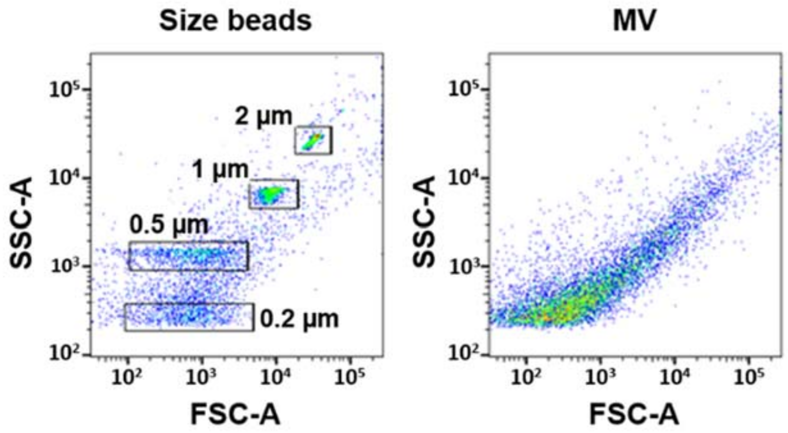

Figure S2. Size characterisation of PC-derived MV. Size profile of PC MV characterised by forward scatter (FSC-A) vs. side scatter (SSC-A) flow cytometry in comparison to a series of beads of known size, including 0.2 µm, 0.5 µm, 1 µm and 2 µm.

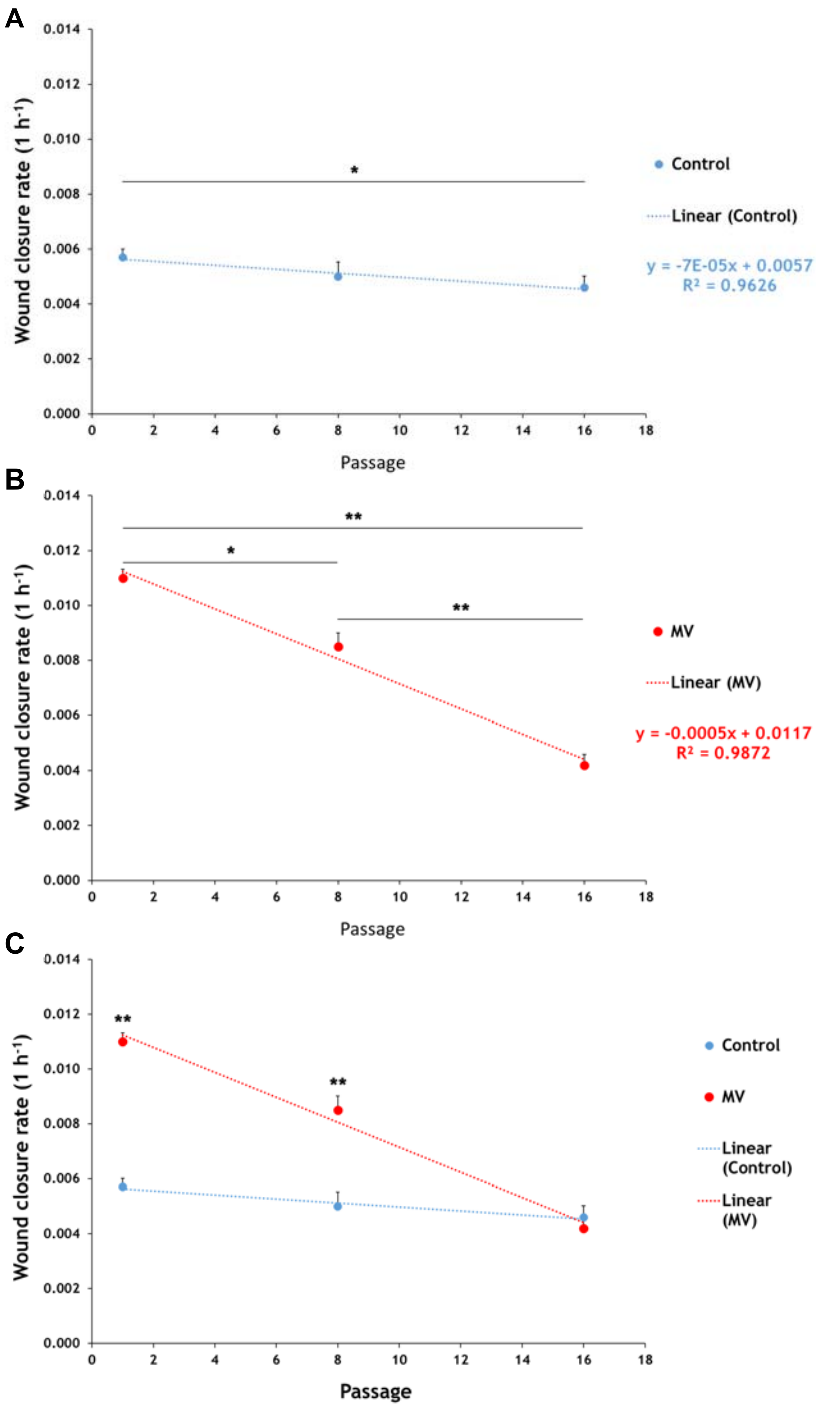

Figure S3. Wound closure rate with increasing cellular age. (**A**) Physiological wound closure rate in HDF-MSC mixed cultures of increasing cellular age. Data are presented as mean ± SD; one-way ANOVA with Tukey's HSD test (N=3). (**B**) Effect of MV administration during wound repair in HDF-MSC co-cultures of increasing cellular age. Data are presented as mean ± SD; one-way ANOVA with Tukey's HSD test (N=3). (**C**) Combined. MV administration (red). PBS administration instead of MV (blue). Data are presented as mean ± SD; t test (N=3). *p<0.05, **p<0.01.

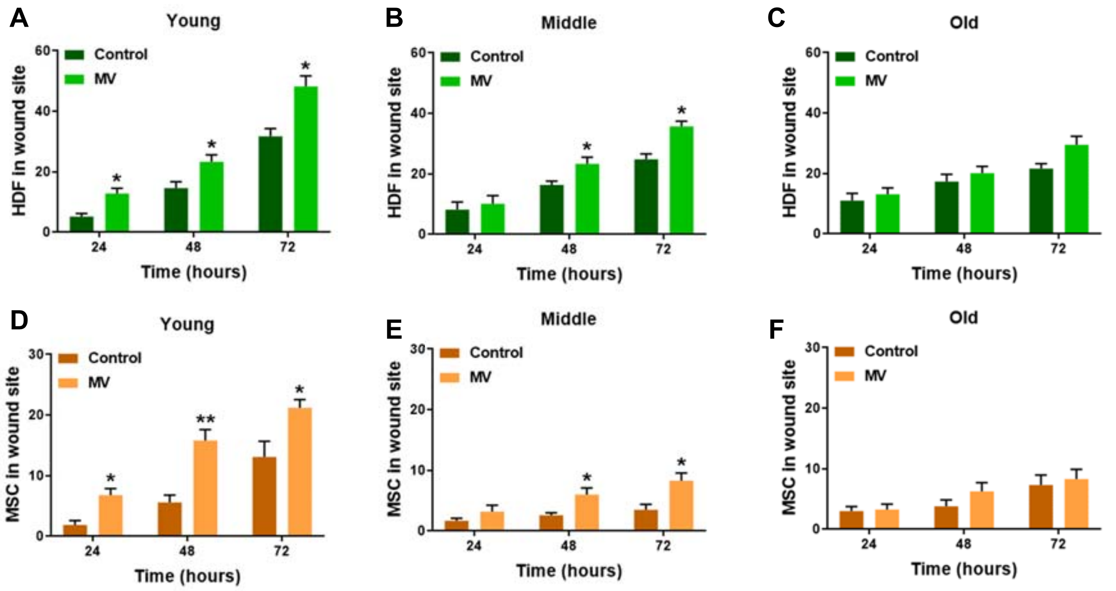

Figure S4. Quantification of HDF and MSC migration into the wound site. Effect of MV administration on HDF migration into the wound site; 24, 48 and 72 hours after wounding in (**A**) young, (**B**) middle, and (**C**) old HDF-MSC co-cultures. Data are presented as mean ± SD; t test (N=3). Effect of MV administration on MSC migration into the wound site; 24, 48 and 72 hours after wounding in (**D**) young, (**E**) middle, and (**F**) old HDF-MSC co-cultures. Data are presented as mean ± SD; t test (N=3). *p≤0.05, **p≤0.01.

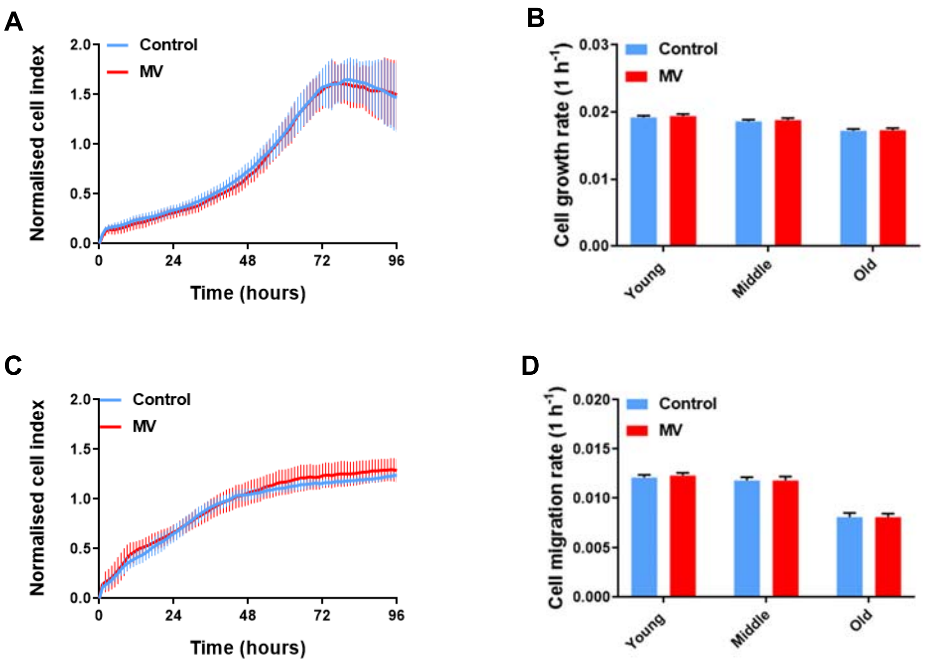

Figure S5. The proliferative and migratory capacity of HDF-MSC co-cultures declines with cellular age, while MV do not affect these processes in the absence of cellular damage. (**A**) RTCA proliferation assay on a young HDF-MSC co-culture. Data are presented as mean ± SD (n=6). (**B**) Cell growth rate of HDF-MSC co-cultures with cellular age. Data are presented as mean ± SD; t test (N=3). (**C**) RTCA migration assay on a young HDF-MSC co-culture. Data are presented as mean ± SD (n=6). (**D**) Cell migration rate of HDF-MSC co-cultures with cellular age. Data are presented as mean ± SD; t test (N=3).

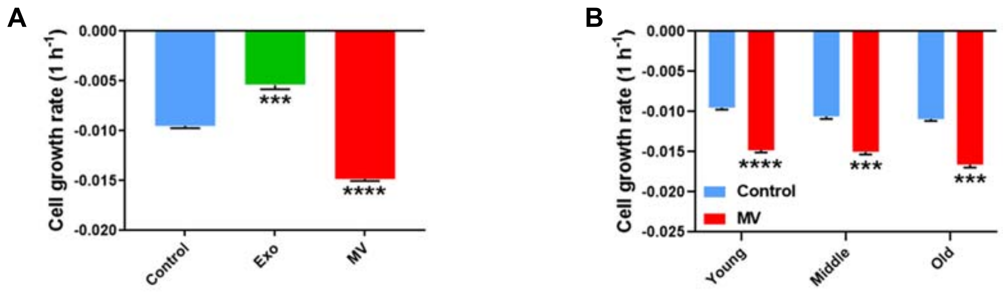

Figure S6. Rate of cell growth in HDF-MSC genotoxicity assays from 0 to 24 hours. (**A**) Cell growth rate of young HDF-MSC co-cultures treated with MV and Exo, 24 hours after uraemic serum addition. Data are presented as mean ± SD; One-way ANOVA with Tukey's test (N=3). (**B**) Effect of MV administration during the first 24 hours of genotoxic stress in HDF-MSC co-cultures of increasing cellular age. Data are presented as mean ± SD; t test (N=3). ***p≤0.001, ****p≤0.0001.

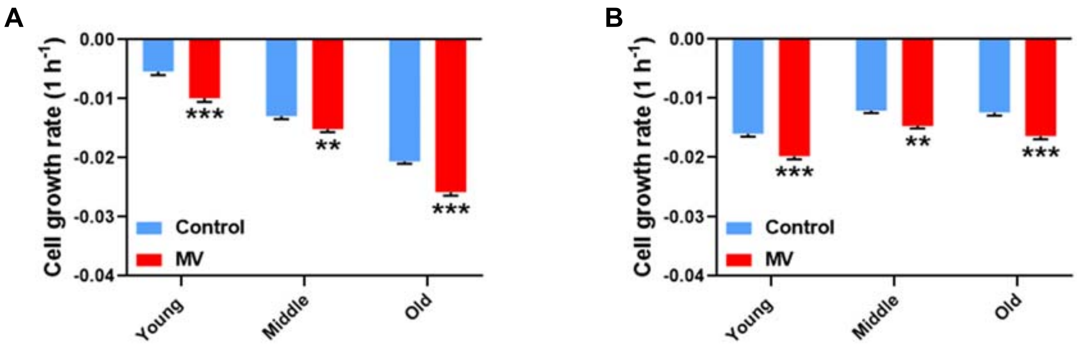

Figure S7. Rate of cell growth in HDF-VSMC and HDF-AAA VSMC genotoxicity assays from 0 to 24 hours. (**A**) Effect of MV administration during the first 24 hours of genotoxic stress in HDF-VSMC co-cultures of increasing cellular age. Data are presented as mean ± SD; t test (N=3). (**B**) Effect of MV administration during the first 24 hours of genotoxic stress in HDF-AAA VSMC co-cultures of increasing cellular age. Data are presented as mean ± SD; t test (N=3). **p≤0.01, ***P≤0.001.

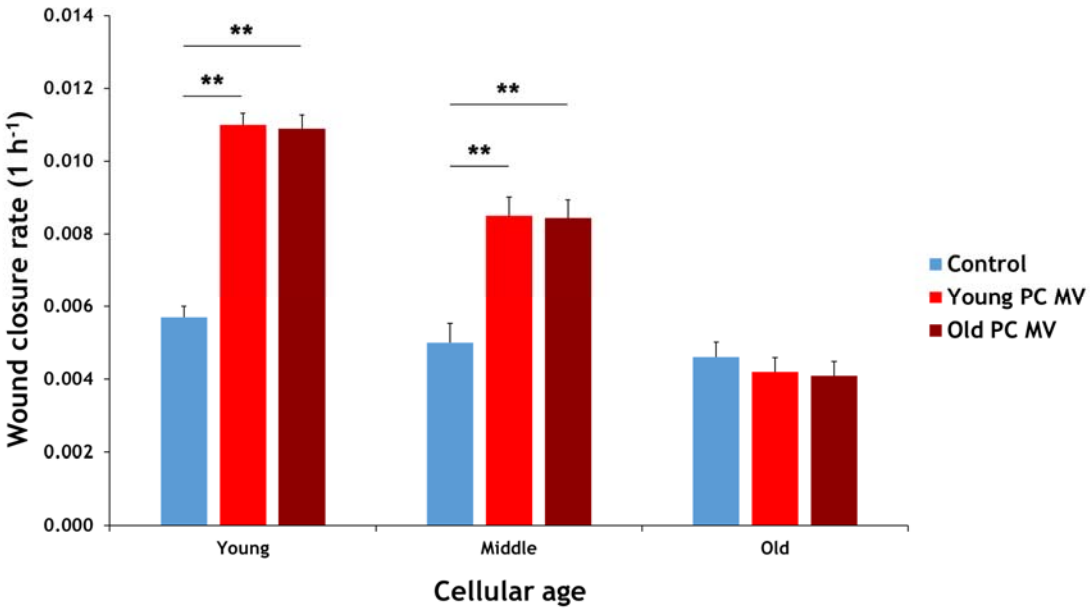

Figure S8. Effect of young versus old cellular age Pathfinder Cell MV administration during wound repair in HDF-MSC co-cultures of increasing cellular age. MVs derived from young Pathfinder Cells [passage 1-4 (cell duplications 1-12)] or from old Pathfinder Cells [passage 17 (cell duplications 51)] were tested for efficacy on HDF-MSC wound healing assays. No differential effects on efficacy were observed relative to donor cell replication age. PBS was administered instead of MVs for controls. Data are presented as mean ± SD; One-way ANOVA with Tukey's test (N=3). ** corresponds to $p<0.01$.
